# Monocytes, particularly nonclassical ones, lose their opsonic and nonopsonic phagocytosis capacity during pediatric cerebral malaria

**DOI:** 10.3389/fimmu.2024.1358853

**Published:** 2024-05-21

**Authors:** Bertin Vianou, Jade Royo, Sébastien Dechavanne, Gwladys I. Bertin, Akadiri Yessoufou, Sandrine Houze, Jean-François Faucher, Agnes Aubouy

**Affiliations:** ^1^ UMR152 PHARMADEV, IRD, UPS, Toulouse University, Toulouse, France; ^2^ Clinical Research Institute of Benin (IRCB), Abomey Calavi, Benin; ^3^ UMR261 Mère et Enfant en Milieu tropical (MERIT), Université Paris Cité, IRD, Paris, France; ^4^ Cell Biology and Physiology Laboratory, Abomey Calavi University (UAC), Abomey Calavi, Benin; ^5^ French Malaria Reference Center, Assistance Publique - Hôpitaux de Paris (APHP), Bichat Hospital, Paris, France; ^6^ Parasitology Laboratory, Assistance Publique - Hôpitaux de Paris (APHP), Bichat-Claude-Bernard Hospital, Paris, France; ^7^ Infectious Diseases and Tropical Medicine Department, Limoges University Hospital, Limoges, France; ^8^ Unité Mixte de Recherche (UMR) 1094 EpiMaCT, Inserm, Limoges University Hospital, Limoges University, Limoges, France

**Keywords:** cerebral malaria, monocyte, nonclassical monocytes, phagocytosis, children, Benin, flow cytometry

## Abstract

**Introduction:**

Innate immunity is crucial to reducing parasite burden and contributing to survival in severe malaria. Monocytes are key actors in the innate response and, like macrophages, are plastic cells whose function and phenotype are regulated by the signals from the microenvironment. In the context of cerebral malaria (CM), monocyte response constitutes an important issue to understand. We previously demonstrated that decreased percentages of nonclassical monocytes were associated with death outcomes in CM children. In the current study, we postulated that monocyte phagocytosis function is impacted by the severity of malaria infection.

**Methods:**

To study this hypothesis, we compared the opsonic and nonopsonic phagocytosis capacity of circulant monocytes from Beninese children with uncomplicated malaria (UM) and CM. For the CM group, samples were obtained at inclusion (D0) and 3 and 30 days after treatment (D3, D30). The phagocytosis capacity of monocytes and their subsets was characterized by flow cytometry and transcriptional profiling by studying genes known for their functional implication in infected-red blood cell (iRBC) elimination or immune escape.

**Results:**

Our results confirm our hypothesis and highlight the higher capacity of nonclassical monocytes to phagocyte iRBC. We also confirm that a low number of nonclassical monocytes is associated with CM outcome when compared to UM, suggesting a mobilization of this subpopulation to the cerebral inflammatory site. Finally, our results suggest the implication of the inhibitory receptors LILRB1, LILRB2, and Tim3 in phagocytosis control.

**Discussion:**

Taken together, these data provide a better understanding of the interplay between monocytes and malaria infection in the pathogenicity of CM.

## Introduction

Malaria remains one of the most common vector-transmitted diseases, leading to a high morbidity and mortality rate. In 2022, there were roughly 249 million cases of malaria in Africa, resulting in 608,000 deaths. Children under 5 accounted for about 80% of all malaria deaths in this region (1). Among the five species that infect humans, *Plasmodium falciparum* is by far the species most responsible for complications (2). Cerebral malaria (CM) is the major lethal complication of *P. falciparum* infection, in addition to severe malarial anemia (SMA). This neurological syndrome occurs in about 1% of infections and is responsible for more than a quarter of malaria deaths annually among children under 5 years old in sub-Saharan Africa (3).

CM is a fatal neurological complication of *P. falciparum* malaria characterized by brain tissue hemorrhages and the accumulation of infected red blood cells (iRBCs) and immune cells in brain microvessels, which can lead to blood–brain barrier (BBB) disruption and death (4). Although sequestration is thought to be the hallmark of CM, the mechanisms involved in the pathophysiology are still incompletely understood. Over the past 15 years, it has become clearer that monocytes play an important role in protective immunity against malaria. However, while activation of the innate immune response is necessary to control blood-stage infection, excessive activation of monocytes has also been implicated in the pathogenesis of severe disease (5, 6).

Based on levels of expression of CD14 (LPS coreceptor) and CD16 (low-affinity Fcγ receptor III), circulating human monocytes are a heterogeneous population comprising three subsets called classical, intermediate, and nonclassical monocytes. Classical monocytes are the most predominant in homeostatic conditions (around 87% in healthy children (7)) and are defined by high expression levels of CD14 on their surface and no surface expression of CD16 (CD14^+^CD16^–^). The intermediate monocytes are characterized by high surface expression of CD14 and CD16 (CD14^+^CD16^+^), whereas the nonclassical monocytes express very low expression of surface CD14 and high levels of CD16 (CD14^low^CD16^+^) (8, 9). It has been shown that the proportions of these subpopulations vary according to the severity of the malaria infection. Acute uncomplicated malaria (UM) was associated with an expansion of intermediate CD14^+^CD16^+^ monocytes (10), while our team reported decreased proportions of circulating nonclassical monocytes in children with CM and in children who died from CM or SMA (11).

With regard to the functional aspect of monocyte subpopulations, data suggest differences in the subset phagocytosis capacity (10). A previous phagocytic assay performed *in vitro* revealed that intermediate CD14^+^ CD16^+^ monocytes were the most effective subset for phagocytosis of *Plasmodium vivax*-infected reticulocytes (12) and complement-opsonized *P. falciparum* iRBCs (13). Garcia-Senosiain et al. showed that nonclassical monocytes were the most efficient subpopulation for phagocytosis of opsonized merozoites. However, most phagocytosis was accomplished by the less efficient ones, the classical monocytes, due to their very high proportion (14). Importantly, it was previously shown that opsonic phagocytosis of merozoites strongly contributes to protection against malaria (15), thereby strengthening the value of our study.

In the current study, we postulated that monocyte phagocytosis function is impacted by the severity of malaria infection. We then compared the opsonic and nonopsonic phagocytosis capacity of circulating monocytes from Beninese children with UM and CM. For the CM group, samples were obtained at inclusion (D0) and 3 and 30 days after treatment (D3, D30). The phagocytosis capacity of monocytes and their subsets was characterized by flow cytometry and transcriptional profiling by studying genes known for their functional implication in iRBC elimination or immune escape. Our results confirm our hypothesis and highlight the higher capacity of nonclassical monocytes to phagocytose iRBC. We also report correlations between the phagocytosis capacity of monocytes and the expression level of genes implicated in phagocytosis mechanisms.

## Materials and methods

### Study design and participants

The study is part of the NeuroCM project, whose goal is to identify the causative factors of neuroinflammation in the context of CM. The NeuroCM project protocol was previously described (16). Briefly, patients were included from March to December 2018. Children were included if they were between 2 months and 6 years old and suffered from CM or UM. Children with CM were enrolled in two reference hospitals from southern Benin: the “Centre Hospitalier Universitaire Mère et Enfant Lagune” (CHU-MEL) in Cotonou and the “Centre Hospitalier Universitaire de Zone d’Abomey Calavi/Sô-Ava” (CHUZAS) in Abomey-Calavi, located 25 km north of Cotonou. UM recruitment was carried out in the Sô-Ava district. CM was defined as (1) deep coma (Blantyre Coma Score ≤ 2) (2), *P. falciparum* infection confirmed by microscopy, and (3) no other known cause of coma (e.g., acute bacterial meningitis, coma related to hypoglycemia reversed by glucose infusion, status epilepticus, preexisting neurological disease, traumatic or toxic coma). UM was defined as (1) fever at inclusion or within 24 h before, (2) no clinical or biological sign of severe malaria as defined by the WHO (17), (3) no danger signs and no other obvious cause of fever, and (4) *P. falciparum* parasitemia between 1,000 and 500,000 parasites per microliter. A negative HIV rapid diagnostic test was also required for children to participate in the study. For the present study of monocyte functionality, we used 19 patients per group, adjusted for age and gender.

### Study approval

Informed consent was obtained from all participants’ parents or their guardians. Ethics approval for the NeuroCM study was obtained from the Comité National d’Ethique pour la Recherche en santé of Benin (n°67/MS/DC/SGM/DRFMT/CNERS/SA; 17 October 2017). The study has also been approved by the Comité consultatif de déontologie et d’éthique of Institut de Recherche pour le Développement (IRD; 24 October 2017).

### Clinical follow-up and blood sampling

As described elsewhere (18), CM children were followed from inclusion (D0) to 30 days after inclusion (D30), including daily clinical monitoring until hospital discharge and a final clinical check at D30. Children presenting with UM were seen only at inclusion (D0). Peripheral venous blood was collected in a vacutainer tube containing EDTA and processed within 4 h, at D0 for both children groups and also at D3 and D30 for the CM children. As detailed before, blood samples were processed for research, hematologic, biochemistry, and microbiological analyses. *P. falciparum* infection was confirmed by real-time PCR, and microbiological coinfections were ruled out through large biological investigations based on blood culture and bacterial culture of cerebrospinal fluid (see details in reference 17).

### *P. falciparum* culture

Asexual stages of *P. falciparum* strain FCR3 were maintained in 0 Rh+ erythrocytes and reduced to 5% hematocrit in flasks containing RPMI medium supplemented with 0.5% AlbuMAX II, 200M-glutamine, 25 mM HEPES, 10 g/ml gentamicin (all purchased in ThermoFischer, Illkirch, France), 0.2 mM hypoxanthin (Merck, Saint-Quentin-Fallavier, France), and 2% human male AB serum (Biowest, Nuaillé, France). The flasks were then gassed with the nitrogen mixture (N_2_ 92.5%, O_2_ 2%, and 5.5% CO_2_) and incubated at 37°C between 18 and 24 h until parasites in mature forms (late trophozoites and schizonts) were predominant (> 50%). Parasite cultures were enriched for knob-expressing strains using 0.75% gelatin sedimentation every 2 weeks.

### Opsonic and nonopsonic phagocytosis

Patient blood samples were processed to isolate peripheral blood mononuclear cells (PBMCs) using Ficoll-Paque PLUS Media (GE Healthcare^®^, Chicago, IL, USA) before cryopreservation. iRBCs at late stages were enriched using MACS magnetic columns (Miltenyi Biotec^®^, Bergisch Gladbach, Germany); 10^7^ iRBCs/ml were labeled with 5 μM CFSE (CellTrace CFSE, ThermoFisher Scientific) for 20 min at 37°C with a shake and washed twice.

For nonopsonic phagocytosis, CFSE-labeled iRBCs (2.10^6^ iRBCs/ml) were incubated with thawed PBMCs (concentration: 2.10^6^ cells/ml) at a 10:1 ratio of iRBCs:monocytes in cell culture medium (RPMI-1640 with 2 mM l-glutamine, 10% FBS). CFSE-labeled iRBCs incubated with THP1 cells were used as positive controls. Since iRBC purification shows day-to-day variations in yield after magnet purification, the number of residuals uninfected red blood cells (uRBCs) remaining after MACS enrichment was calculated, and this amount of uRBCs was incubated with participant’s thawed PBMCs as blank control for each nonopsonic phagocytosis assays.

For opsonic phagocytosis, pooled plasma from eight malaria-immune Beninese adults was used for opsonization. CFSE-labeled iRBCs (2.10^6^ IRBCs/ml) were opsonized with heat-inactivated plasma (at a final plasma concentration of 1:10) for 1 h and then washed in RPMI-HEPES. Opsonized CFSE-labeled iRBCs were incubated as before with thawed PBMCs. Opsonized CFSE-labeled iRBCs incubated with THP1 were used as a positive control. Residual opsonized CFSE-labeled uRBCs incubated with participants’ PBMCs were used as blank controls.

The cells were incubated in polypropylene tubes for 40 min at 37°C in 5% CO_2_ on an orbital shaker. After incubation, the cells were placed on ice and washed in PBS with 2 mM EDTA. PBMCs were stained for 15 min in the dark with LIVE/DEAD Viobility 405/520 Fixable Dye followed by 20 min at 4°C staining with APC-Vio770-labeled anti-CD16 (REA423; Miltenyi Biotec) and APC-labeled anti-CD14 (REA599; Miltenyi Biotec). Cells were fixed using PFA 4% and subjected to flow analysis using a Fortesa flow cytometer (BD Biosciences, Rungis, France). A minimum of 20,000 CD14^+^ cells was acquired. The percentage of phagocytosis was calculated according to the monocytes with CFSE fluorescence. Geometric means of CFSE-mean fluorescence intensity (MFI) were also analyzed. To compare the phagocytosis capacity of monocytes between follow-up points and avoid day-to-day experimental variation in phagocytosis assays, opsonic and nonopsonic phagocytosis assays were run in the same-day assay for each sample from acute CM (CM D0) and their own samples collected 3 days (CM D3) and 30 days (CM D30) following treatment and their matched UM.

### Monocyte transcriptional profiles

Thawed PBMCs (500,000/well) were plated in 48-well plates in culture medium and allowed to adhere in a 5% CO_2_ container at 37°C for 2 h. Nonadherent cells were removed by thorough washing with RPMI‐1640. Adherent cells were lysed with 100 µl of lysis solution, and total RNA was extracted using the RNAqueous^®^-Micro Kit (Invitrogen, Illkirch, France) following the manufacturer’s instructions. Genes coding for Fc-gamma receptors (FcγRI, also called CD64, and FcγRIII, also called CD16), complement receptors (CR1, also called CD35 and CR3, also called CD11b), pattern recognition receptors (CD36, CD206, TLR2, CD163), and inhibitory immune checkpoint receptors (LILRB1, LILRB2, T-cell immunoglobulin- and mucin-domain-containing molecule 3 (Tim3)) were amplified by qPCR on Quant Studio 5 (Applied Biosystems, Les Ulis, France). PCR conditions were 50°C for 2 min, 95°C for 10 min, then 40 cycles (95°C for 15 seconds, 60°C for 60 s), followed by a dissociation step from 65°C to 95°C. Using β-actin as a housekeeping gene and monocytes from healthy children as a control sample, the Pfaffl method was used to calculate the relative expression of monocyte transcripts. Primer sequences are summarized in **Supplementary Table 1**.

### Plasma cytokines

Using Luminex technology, plasma levels of nine pro- and anti-inflammatory cytokines were simultaneously measured. The Human Premixed Multi-Analyte Kit (LXSAHM, R&D Systems, Lille, France) was used according to the manufacturer’s recommendation by the Anexplo platform (Genotoul, Toulouse, France).

### Statistical analysis

Statistical analysis was performed on GraphPad Prism software (version 10.1.0). For descriptive analysis, quantitative variables were presented as the means ± standard deviations or medians of interquartile ranges, and qualitative variables were presented as frequencies (percentages). The Kruskal–Wallis test with Dunn’s test for multiple comparisons and the Mann−Whitney *U* test were used to compare the percentages of phagocytosis and gene ratio between the four groups (CM D0, CM D3, CM D21, and UM). To evaluate correlations between the phagocytosis capacity of monocytes and gene expression level, the nonparametric Spearman correlation test was used. Differences were considered significant at *p* < 0.05.

## Results

### Clinical and blood count characteristics of the children studied

The clinical and biological characteristics of the children studied are presented in **Table 1**, according to their clinical group. Each group comprised 14 girls and five boys with a mean age around 49 months for both groups. Axillary temperature and parasitemia were also similar in the two groups. All children in the CM group were characterized by low hemoglobin levels with a mean of 5.7 g/dl (± 2.0). Higher absolute counts of leukocytes, lymphocytes, neutrophils, and eosinophils were related to malaria severity (*p* = 0.0024, *p* = 0.03, *p* = 0.001, and *p* = 0.02, respectively), in contrast to basophils and thrombocytes, whose low levels were associated with CM. The absolute counts of monocytes were slightly higher in the UM group, but this difference was not significant, allowing us to exclude any possible relationship between our results and the monocyte counts of our patients. For biochemistry factors, glucose and creatinine levels were not related to the clinical group.

**Table 1:** Sociodemographic characteristics of the study population.

	CM (*n* = 19)	UM (*n* = 19)	*p*−value^a^
Gender (*n*/*n* total (%))			
Woman	14/19 (73.7)	14/19 (73.7)	> 0.9999
Man	5/19 (26.3)	5/19 (26.3)	
Age in months (mean (± SD))	48.73 (± 11.5)	49.58 (± 11.9)	0.46
Man	50.3 (± 9.8)	50.6 (± 10.6)	0.73
Woman	48.2 (± 11.7)	49.2 (± 12.8)	0.47
Weight (kg; mean (± SD))	13.6 (± 2.7)	14.7 (± 2.8)	0.17
Axillary temperature (°C; mean (± SD))	38.36 (± 1.2)	37.92 (± 1.2)	0.42
*P. falciparum* density/μl of blood (median; IQR)	35,700 (3,343–137,983)	19,776 (6,676–86,180)	0.77
Mean hemoglobin level (g/dl (± SD))	5.7 (± 2.0)	8.9 (± 1.7)	**< 0.0001**
Hemoglobin level < 11 (*n*/*n* total (%))	19/19 (100)	17/19 (89.5)	**< 0.0001**
Hematocrit (mean (± SD))	16.7 (± 6.5)	27.9 (± 4.4)	**< 0.0001**
Leucocytes × 10^3^/μl (mean (± SD))	13.7 (± 10.6)	8.04 (± 5.8)	**0.002**
Lymphocytes × 10^3^/μl (mean (± SD))	4.7 (± 4.7)	2.3 (± 1.2)	**0.03**
Monocytes × 10^3^/μl (mean (± SD))	0.6 (± 0.5)	0.9 (± 0.7)	0.08
Neutrophils × 10^3^/μl (mean (± SD))	8.2 (± 6.1)	4.7 (± 5.3)	**0.001**
Eosinophils × 10^3^/μl (mean (± SD))	0.2 (± 0.2)	0.05 (± 0.1)	**0.02**
Basophils × 10^3^/μl (mean (± SD))	0	0.03(± 0.04)	**0.02**
Thrombocytes × 10^3^/μl (mean (± SD))	99.8 (± 52.7)	210.6 (± 151.5)	**0.002**
Glucose (g/L; mean (± SD))	1.08 (± 0.8)	0.9 (± 0.2)	0.93
Creatinine (mg/L; mean (± SD))	4.1 (± 2.1)	3.2 (± 0.3)	0.13
Blantyre score (*n*)			
0	0	NA	
1	4	NA	
2	15	NA	

a Statistical differences between clinical groups were calculated using the Mann−Whitney U test for quantitative variables, and Pearson’s Chi^2^ test for qualitative variables. p-values < 0.05 are written in bold. SD, standard deviation; IQR, interquartile range; NA, nonattributable.

### A decrease in the nonclassical monocyte subset in peripheral blood is associated with acute CM

**Figure 1:**
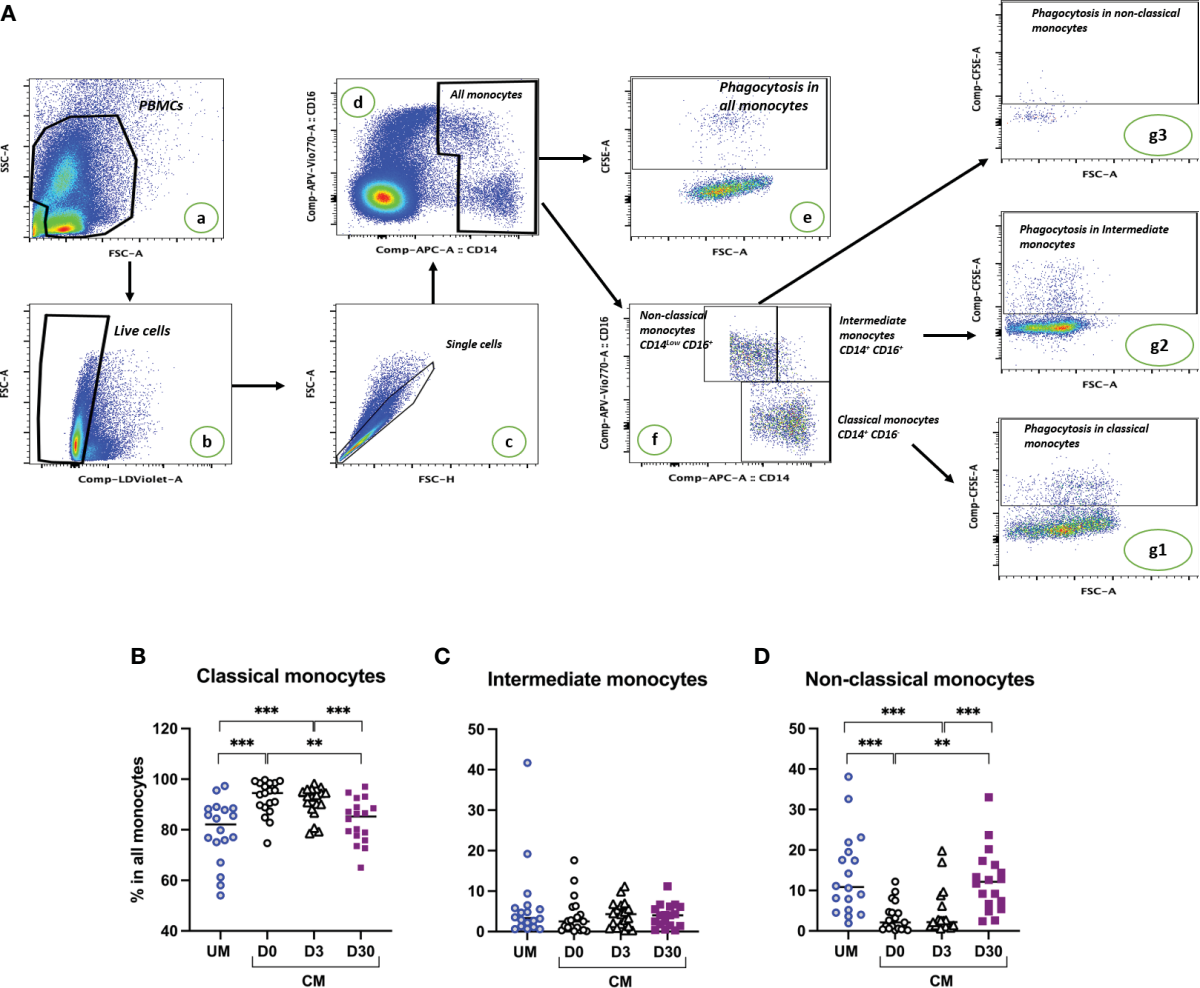
Cerebral malaria (CM) resolution is associated with a decrease in the classical monocyte subset and an expansion of the nonclassical subset. **(A)** Gating strategy to determine proportions of monocyte subsets and phagocytic monocytes. PBMCs were selected (a), dead cells were excluded based on live dead staining (b), doublets were excluded (c), and monocyte populations were gated based on CD14 and CD16 staining (d). The phagocytosis proportion of all monocytes was determined based on CFSE labeling (e). Among monocytes, proportions of the three subsets were determined based on CD14 and CD16 expression levels (f). Phagocytosis proportion in each subset was determined based on CFSE labeling (g1, g2, g3). Proportions of classical **(B)**, intermediate **(C)**, and nonclassical monocytes **(D)** are shown for children presenting uncomplicated malaria (UM) or CM at inclusion (D0) and 3 and 30 days after inclusion (D3, D30). Proportions were compared by Mann–Whitney *U* test; ^***^ *p* < 0.001; ^**^ *p* < 0.01.

Based on the expression level of CD14 and CD16 on their surfaces, classical, intermediate, and nonclassical subsets were identified by flow cytometry (**Figure 1A**). The percentages of monocyte subsets were compared according to the clinical group (CM, UM) and the time of follow-up for the CM children (D0, D3, D30). Compared to UM, percentages of classical monocytes appear to be higher during acute CM (**Figure 1B**). Although the percentages of classical monocytes appeared to increase for CM at D0 and D3, they remained around the values of the physiological state (median percentage for CM D0 = 94.5%, for CM D3 = 93.2%, **Figure 1B**). The percentages of intermediate monocytes were similar in both clinical groups and during follow-up in CM (**Figure 1C**). Conversely, the percentage of nonclassical monocytes in CM at inclusion was far below that of the UM group (*p* < 0.0001, **Figure 1D**). Interestingly, the respective percentages of classical and nonclassical monocytes gradually returned at D3 and then D30 to levels comparable to those in the UM group, with levels statistically different from those of CM at inclusion (*p* < 0.0001 and *p* = 0.0002, respectively) (**Figures 1B, D**).

### Acute CM is associated with impaired monocyte phagocytosis of iRBCs

**Figure 2:**
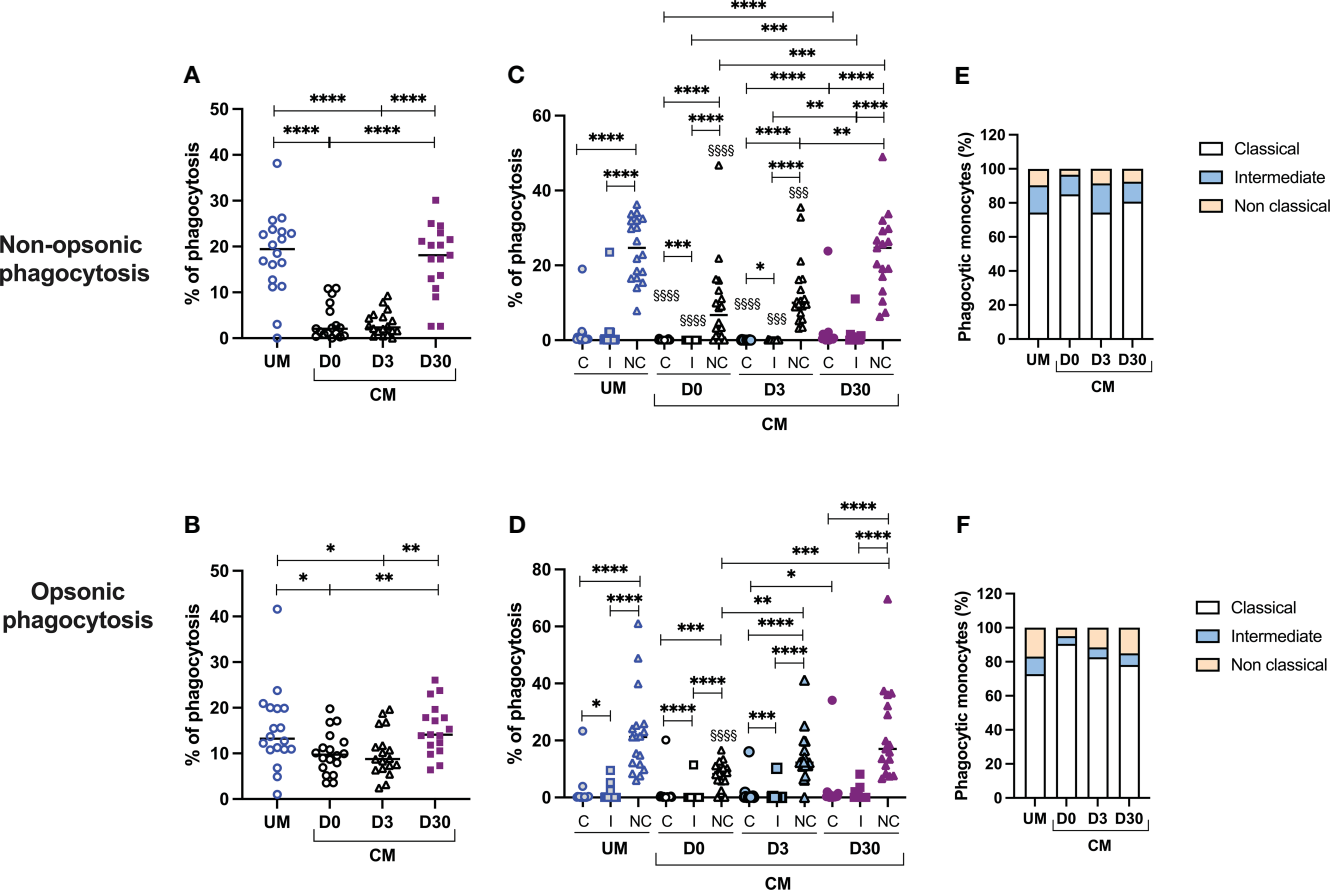
Decreased phagocytosis activity of monocytes, and particularly nonclassical ones, is associated with cerebral malaria (CM). Proportions of nonopsonic phagocytosis **(A)** and opsonic phagocytosis **(B)** were calculated among total monocytes. Proportions of nonopsonic and opsonic phagocytosis in each monocyte subset **(C, D)** were evaluated for each monocyte subset as the ratio of the number of monocytes in the subpopulation having phagocytosed to the total number of monocytes in the subpopulation. Finally, the respective contribution of each monocyte subset to the phagocytosis capacity of total monocytes was calculated in percentage **(E, F)**. ^§^Comparisons of the level of phagocytosis of each monocyte subpopulation in the CM group with the corresponding subpopulation in the UM group. UM, uncomplicated malaria; D0, day of inclusion; D3, day 3 after inclusion; D30, day 30 after inclusion. Proportions were compared by the Mann–Whitney *U* test. One symbol: *p* < 0.05, two symbols: *p* < 0.01, and three symbols: *p* < 0.001, and four symbols: *p* < 0.0001.

Monocytes are known for their ability to reduce parasite burdens through phagocytosis. We postulated that CM could modify the phagocytic capacity of monocytes. Nonopsonic and opsonic phagocytic activities of the participants’ monocytes were assessed during acute UM, acute CM (CM D0), and 3 and 30 days following inclusion for the CM group (CM D3 and D30) (**Figure 2**). Interestingly, opsonic and nonopsonic phagocytic activities of total monocytes (**Figures 2A, B**) were decreased during acute CM (CM D0) and 3 days after inclusion (CM D3) compared to that of UM monocytes or CM monocytes at D30. In the CM group, both phagocytic capacities were recovered at D30 to reach a level similar to that of UMs. The difference was particularly marked for nonopsonic phagocytosis (median values of 2.05% phagocytosis for CM D0 vs. 2.35% for CM D3 and 18.1% for CM D30, **Figure 2A**). **Supplementary Figure 1** shows the same analysis using CFSE MFIs. Similar results were obtained for nonopsonic phagocytosis, whereas no significant differences in opsonic phagocytosis capacity were found through this approach, underlying the strength of our results for nonopsonic phagocytosis.

To go further, we then measured the capacity of each monocyte subset to phagocytose iRBC according to their clinical group and time of sampling (**Figures 2C, D**). First, it is interesting to note that nonclassical monocytes were the most capable of nonopsonic and opsonic phagocytosis, compared with classical and intermediate monocytes in UM and CM children (for nonopsonic phagocytosis in UM and CM: nonclassical vs. intermediate and classical monocytes, *p* < 0.0001; for opsonic phagocytosis in UM: nonclassical vs. intermediate and classical monocytes, *p* < 0.0001; for opsonic phagocytosis in CM: nonclassical vs. intermediate and classical monocytes, *p* < 0.0001 and *p* = 0.0002, respectively; **Figures 2C, D**). This observation was also true in the CM group during follow-up (*p* < 0.0001 for all comparisons). Next, we compared the phagocytosis capacity of each subpopulation between clinical conditions (CM vs. UM at D0) and between the times of sampling (D0 vs. D3 vs. D30). For nonopsonic phagocytosis, for the CM D0 and CM D3 groups, a lower capacity of the monocytes of the three subpopulations was observed compared with the respective subpopulations of the UM group (**Figure 2C**). For opsonic phagocytosis, such a comparison was true only for nonclassical monocytes from CM D0 compared to those from UM (**Figure 2D**). For nonopsonic phagocytosis, the monocyte capacity of phagocytosis for each subset was recovered at D30 in the CM group, without any difference between D0 and D3 capacities (**Figure 2C**). For opsonic phagocytosis, nonclassical monocytes also recovered their capacity to phagocytes, with difference observed between D0 and D3 (median values of 8.2% vs. 12.3%, *p* = 0,006) and D0 and D30 (median values of 8.2% vs. 17.0%, *p* = 0.0003).

The last two graphs show the respective contribution of each subpopulation to the phagocytosis capacity of total monocytes (**Figures 2E, F**). We observed that the classical monocytes, as well as the less efficient phagocyte iRBC, contributed most to iRBC nonopsonic and opsonic phagocytosis. This higher contribution was true in the UM and CM groups and also at D3 and D30 in CM children. This result is due to the much greater proportion of classical monocytes compared with the other two subpopulations in both clinical groups and at each time point (**Figure 1**).

### Monocyte transcriptional profiles and relation with cerebral malaria

Using RT-qPCR, 11 genes implicated in the phagocytosis process were analyzed. Two Fcγ receptors were studied: FcγRI (also called CD64), known for its high-affinity binding capacity to monomeric IgG, and FcγRIII (also called CD16), known for its implication in antibody-dependent cellular cytotoxicity, an important way of iRBC elimination (19, 20). To investigate complement implications, complement receptors CR1 and CR3 expression levels were measured, known for their capacity to recognize opsonins bound to antigens or immune complexes (21, 22). Three scavenger receptors were also studied: CD36, known for its implication in nonopsonic phagocytosis of iRBCs; CD206 (also called Mannose receptor 1), known for its implication in the phagocytosis of pathogens such as *Leishmania* and a lower rate of *P. falciparum*-iRBCs (23) and in the M2-like phenotype; and CD163, known for its implication in the hemoglobin–haptoglobin complex elimination, an important step of detoxification during malaria. TLR2, a toll-like receptor, is also a pattern-recognition receptor as the scavenger receptor. Known for its collaboration with CD36 and CD163 in activating signaling pathways and enhancing iRBC internalization (24), we also studied its expression. Finally, LILRB1, LILRB2, and Tim3 expressions were evaluated as key inhibitory receptors implicated in the balance between parasite immune evasion and immune responses to infectious diseases (25–27).

**Figure 3:**
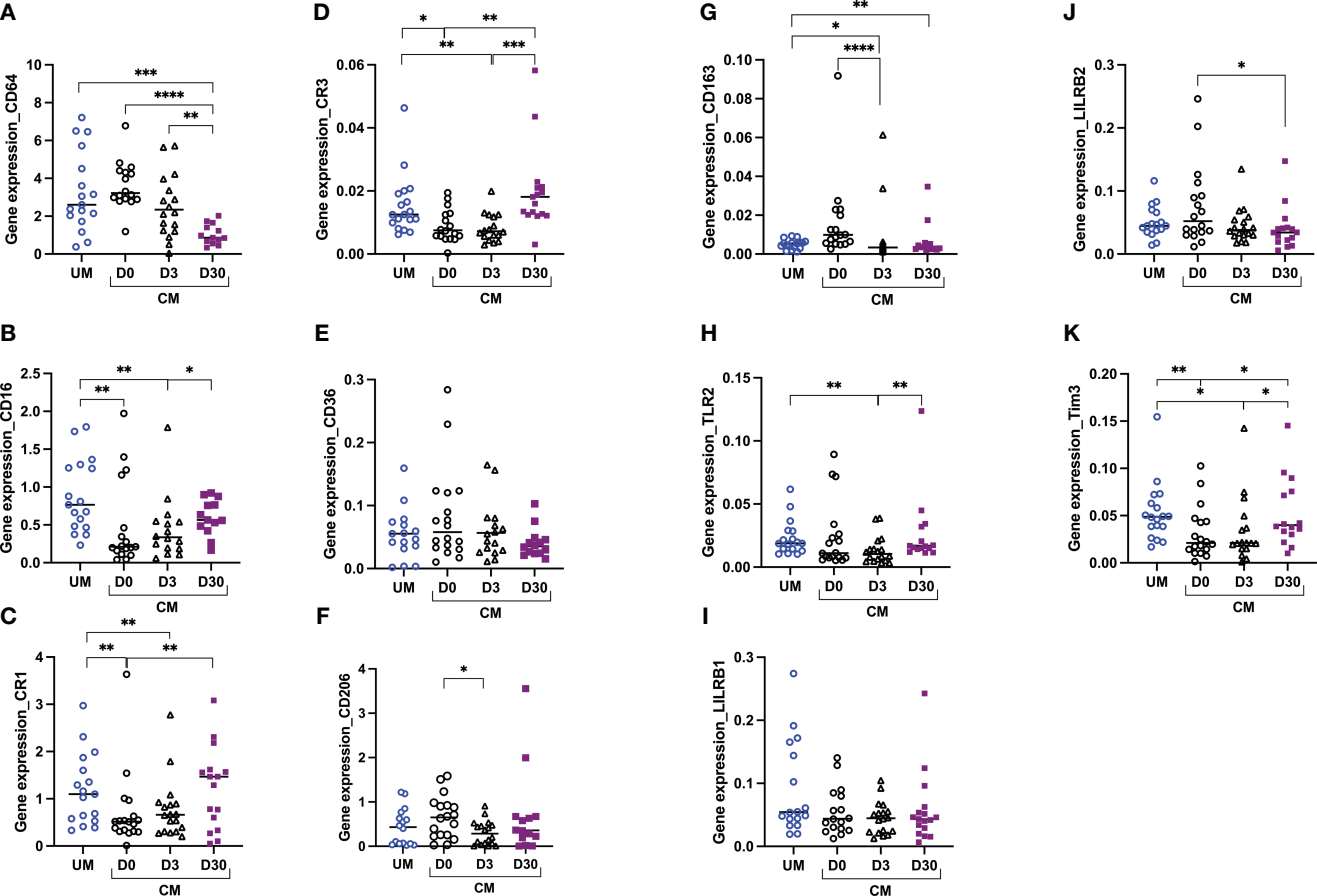
Altered monocyte gene profiles are associated with acute cerebral malaria (CM). CD64 **(A)**, CD16 **(B)**, CR1 **(C)**, CR3 **(D)**, CD36 **(E)**, CD206 **(F)**, CD163 **(G)**, TLR2 **(H)**, LILRB1 **(I)**, LILRB2 **(J)**, and Tim3 **(K)** transcript levels were evaluated by RT-qPCR in total monocytes from children presenting with uncomplicated malaria (UM) or CM at inclusion (D0) and 3 and 30 days after inclusion (D3, D30). Gene expression levels were calculated using the Pfaffl method and are expressed as ratios. Ratios were compared by the Mann–Whitney *U* test; ^****^ *p* < 0.0001; ^***^ *p* < 0.001; ^**^ *p* < 0.01; ^*^ *p* < 0.05.

Monocyte transcriptional profiles were compared between clinical origin (CM and UM) and between times of follow-up in the CM group. **Figure 3** shows the results of gene expression in total monocytes. Compared to UM’s monocytes, monocytes from CM children at D0 expressed lower levels of CD16, CR1, CR3, and Tim3. For these four genes, expression levels were gradually restored at D30 to comparable levels as those of UM’s monocytes (**Figures 3B–D, K**), suggesting an inhibition of these four genes at D0 in the CM group. Although no difference was observed between TLR2 expression at D0 between the CM and UM groups, its expression profile was similar, with an increase between D3 and D30 in the CM group (**Figure 3H**). Conversely, CD64, CD206, CD163, and LILRB2 had decreasing expression from D0 to D30 in the CM group, with comparable expression levels between CM’s and UM’s monocytes at D0, suggesting an activation of these genes in CM’s monocytes at D0 (**Figures 3A, F, G, J**).

**Figure 4:**
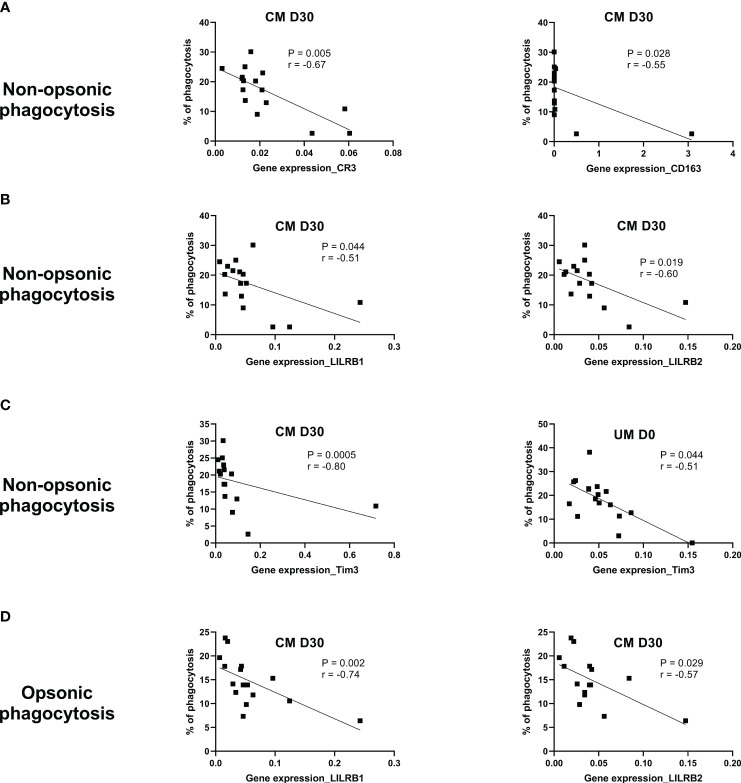
CR3, CD163, LILRB1, LILRB2, and Tim3 gene expression are related to phagocytosis control during cerebral malaria (CM). The Spearman correlation test was used to evaluate the relation between the phagocytosis capacity of monocytes and gene expression level in uncomplicated malaria (UM) and CM groups and during follow-up for the CM group (D3, D30). **(A)** Correlations between CR3 or CD163 expression and nonopsonic phagocytosis of CM’s monocytes (NOP-CM). **(B)** Correlations between LILRB1 or LILRB2 expression and NOP-CM. **(C)** Correlations between Tim3 expression and NOP-CM or NOP-UM. **(D)** Correlation between LILRB1 or LILRB2 expression and opsonic phagocytosis. The significance and strength of the correlation are indicated on each graph by the *p*- and *r* values.

To understand whether the genes studied were involved in the phagocytosis of monocytes from UM and CM patients, the correlation between their expression levels and phagocytosis capacity was assessed. Interestingly, for the CM group, significant correlations were found at D30, only suggesting that phagocytosis at D0 was too disrupted to find any correlation. At D30, the nonopsonic phagocytosis capacity of monocytes from CM children was inversely related to CR3, CD163, LILRB1, LILRB2, and Tim3 expressions, as shown in **Figures 4A–C** (*p* = 0.005, *r* = −0.67; *p* = 0.028, *r* = −0.55; *p* = 0.044, *r* = −0.51; *p* = 0.019, *r* = −0.60; *p* = 0.0005, *r* = −0.80; respectively). For opsonic phagocytosis at D30, such a relation was found only for LILRB1 and LILRB2 (*p* = 0.002, *r* = −0.74; *p* = 0.029, *r* = −0.57) (**Figure 4D**). In the UM group, the capacity of monocytes to phagocyte iRBC by nonopsonic phagocytosis was inversely related to Tim3 expression (*p* = 0.014, *r* = −0.59, **Figure 4C**), but no such relation was found for opsonic phagocytosis. These results suggest the involvement of these five genes in the control of phagocytosis.

### Plasmatic immune markers and relation to monocyte transcriptional profile

To assess the general immune state of the children, we measured plasma cytokines and chemokines in order to study their relations with monocyte transcriptional profiles of immune response inhibitors (LILRB1, LILRB2, and Tim3). **Supplementary Figure 2** reveals that CM children have a proinflammatory profile at D0 compared to D30. Indeed, plasma levels of TNF, IL-6, CXCL9, and CXCL10 were high at inclusion (D0) and gradually decreased until D30 (**Supplementary Figures 2A, B**), whereas CCL17 and CCL22 presented opposite profiles (**Supplementary Figure 2C**). As commonly found during CM, the IL-10 profile, an anti-inflammatory cytokine, was similar to that of proinflammatory cytokines (**Supplementary Figure 2C**). Also, the CXCL5 level, a chemokine implicated in neutrophil migration (28), was lower at D0 compared to D3 and D30, as if neutrophil response was limited at inclusion (**Supplementary Figure 2B**). Plasma CXCL9 levels were inversely related to LILRB2 gene expression at D3 in CM children and positively related at D30 (**Supplementary Figures 3A, B**). At D30, plasma CXCL9 levels were also positively related to Tim3 gene expression (**Supplementary Figure 3C**) in CM children. In UM children, plasma IL-8 and LILRB2 gene expression were negatively correlated (**Supplementary Figure 3D**).

## Discussion

The aim of this study was to assess the functionality of monocytes during CM, based on the hypothesis that CM alters the phagocytosis capacity of monocytes. For this purpose, monocytes from children aged 24 to 71 months and presenting with CM or UM were studied. The children, 19 in each group, were matched on gender and age to avoid bias on these two parameters. Coinfections and nonmalarial comas were also ruled out through blood culture and bacterial culture of cerebrospinal fluid (18). In the CM group, only surviving children were included and were followed 3 and 30 days after inclusion (D3, D30). Furthermore, most of the studies focusing on monocyte functionality were carried out using leukocytes from healthy subjects and lab-cultured parasites, with only the antibodies used for opsonization coming from immune patients (12–15). Our approach has the advantage of working with patients’ monocytes, reflecting the real impact of malaria on the phagocytosis levels of these cells.

We first compared monocyte proportions between the CM and UM groups at D0 and during follow-up for the CMs. We found lower proportions of nonclassical monocytes in the CM group compared to that of UM’s at D0. This proportion remained low at D3 and was restored at D30 to a level similar to that of the UM group. Conversely, the proportion of classical monocytes at D0 was higher in the CM group compared to the UM group at D0 and decreased until D30 to a similar level to that of the UM group. These results are very consistent with previous data published by our team, which also showed a lower proportion of nonclassical monocytes in CM compared with UM at inclusion and an association between this lower proportion and the occurrence of death (11). Our results are also supported by those of Dobbs et al., who found lower levels of nonclassical monocytes in children with UM compared to healthy children of the same age (10). These results suggest that nonclassical monocytes are mobilized during malaria at iRBC sequestration sites, and more specifically in cerebral capillaries in the case of CM. Nonclassical monocytes display patrolling properties and are known to be involved in inflammation resolution during noninfectious pathologies (29). We thus postulated that the decrease in the proportion of nonclassical monocytes in the bloodstream during acute CM observed here could be due to their recruitment to the inflammatory sites in the brain.

Next, to explore the question of monocyte functionality, we assessed the phagocytosis capacity of monocytes from the CM and UM groups. Acute CM was associated with impaired monocyte phagocytosis of iRBCs when compared to UM. Opsonic, and more particularly nonopsonic, phagocytosis, was notably decreased in total monocytes of children presenting with CM at inclusion and 3 days after treatment, compared to their own samples collected 30 days following treatment. This loss of phagocytic capacity could be explained by the digestion of hemozoin by monocytes. Hemozoin, the end product of hemoglobin digestion by parasites, is known for its immunomodulatory activities, including phagocytosis impairment (30, 31). In addition, CD47-SIRPα interactions were shown to negatively regulate macrophage phagocytosis of iRBCs in murine models, and anti-CD47 treatment was found to significantly increase the clearance of iRBCs (32). The CD47-SIRPα axis is also known for its implication on monocyte migration, dendritic cell functions, and maturation (33), suggesting that additional studies are needed to determine the extent to which this axis is involved in the inhibition of phagocytosis. In a recent study, Garcia-Senosian et al. demonstrated that neutrophils are the main phagocytes of iRBC *in vitro* when compared to monocytes (14). In addition, their study reveals that monocytes are more efficient at nonopsonic phagocytosis than at opsonic phagocytosis, which is in line with our results. Indeed, in **Figure 2**, median percentages of phagocytosis were around 20% for nonopsonic phagocytosis in UM and CM D30, versus 12% for opsonic phagocytosis. Moreover, these authors showed that the contribution of monocytes in opsonic phagocytosis of iRBC was related to the degree of antibody opsonization, with monocytes gaining the upper hand over neutrophils at lower levels of opsonizing antibodies at higher plasma dilutions. These results suggest that monocyte response is of high importance in nonimmune subjects, such as children under the age of 5 in malaria-endemic areas.

To date, few studies have investigated the relationship between each monocyte subset, their phagocytosis potential, and their association with severity during *Plasmodium* spp. infection. To determine whether the expanded subset is contributing to protection or pathogenesis and could potentially be used as a therapeutic target, we investigated the phagocytic capacity of each subset of our malaria patients and found that nonclassical monocytes exhibit a higher phagocytic capacity. In agreement with our results, previous studies have reported similar associations. Focusing on monocyte subset phagocytosis capacity, Dobbs et al. found that intermediate and nonclassical monocytes from Kenyan children with acute malaria had greater phagocytic activity than classical monocytes for both opsonic and nonopsonic phagocytosis (10). However, using whole blood from healthy people cocultured with opsonized *P. falciparum* lab lines (iRBCs), Zhou et al. found that intermediate monocytes had greater *P. falciparum* opsonic phagocytic activity than the other monocyte subsets (13). Garcia-Senosian et al. also found a higher capacity of opsonic phagocytosis for intermediate and nonclassical monocytes from healthy subjects compared to classical monocytes, with a nonsignificant superiority of nonclassics. Interestingly, their study also showed that classical monocytes were nevertheless the biggest contributors to opsonic phagocytosis, among the three subpopulations, due to their much greater absolute numbers than the other two subpopulations (14). We obtained the same result for both opsonic and nonopsonic phagocytosis in the two clinical groups studied, UM and CM, and in CM, this was also true during follow-up at D3 and D30 after inclusion.

Low expression of CD16, CR1, CR3, and Tim3 by total monocytes was related to CM outcome when compared to UM, and expression of these markers normalized to similar levels to that of UMs upon recovery. For CD16, CR1, and CR3, three receptors implicated in opsonization, such expression profiles are consistent with the lower opsonic phagocytosis capacity of monocytes that we found and suggest an immunosuppressive effect of CM, as Mandala et al. concluded in their study (34). However, it is surprising that no difference in CD36 expression, the receptor involved in nonopsonic phagocytosis of iRBC, was obtained in our study. Our team has previously shown a link between lower levels of CD36 protein expression and the occurrence of CM and death (11). However, the expression levels of a gene and its protein can be shifted over time. Interestingly, in our study, CR3 expression was inversely related to the nonopsonic phagocytosis capacity of monocytes at D30 in CM children. This result suggests a balance between opsonic and nonopsonic phagocytosis that could be controlled in part by CR3 expression, in the absence or low level of immunity, as in the case of the children in our cohort.

Mandala et al. also studied the surface expression of TLR2 on the total monocytes of Malawian children presenting with UM, severe malarial anemia, and CM upon recovery (4 weeks later). They found a lower expression of TLR2 in the severe malarial anemia and CM groups compared to healthy controls (34). In contrast, Dobbs et al. found elevated surface expression of TLR2 on intermediate monocytes from Kenyan children with acute UM compared to healthy children (10). In our study, TLR2 gene expression in monocytes was similar between CM and UM groups, but a profile of increase was observed from D3 to D30 in the CM group, suggesting inhibition of TLR2 expression during the acute phase of CM and the first days after CM treatment. TLR2 is known for its interaction with *P. falciparum* glycosylphosphatidylinositol (GPI) and the subsequent activation of the inflammatory response (35). Such downregulation could be an actor in the pro- and anti-inflammatory response balance regulation. During the past decade, Tim3 has been shown to be an important immunomodulatory molecule, as LILRB1 and LILRB2, two receptors interacting with HLA-I molecules to negatively modulate effector immune cells (36, 37). It is interesting to note that Tim3 and LILRB2 had opposite gene expression profiles in the monocytes of the CM group over time. While LILRB2 expression appeared to decrease over time, Tim3 expression increased, suggesting a possible balance between these two genes in regulating the immunosuppressive effect. However, LILRB1, LILRB2, and Tim3 were all found to be inversely related to the nonopsonic phagocytosis capacity of monocytes in the CM group at D30. The same relation was found for Tim3, expressed by the UM’s monocytes. These results do not support our first hypothesis and point instead to inhibitory functions for these three receptors in relation to monocyte phagocytosis. Our results also suggest a role for LILRB1 and LILRB2 in the regulation of monocyte opsonic phagocytosis. To learn more about these three inhibitory genes, we tested the correlations between their expression level and plasma cytokine levels, regarded as immune markers of inflammation. The relation found between CXCL9 plasma levels, a chemokine implicated in Th1 response, CD8 T cells, and NK cell trafficking (28) and recently reported for its implication in the pathogenesis of pediatric cerebral malaria (18), and LILRB2 revealed opposite immune mechanisms between D3 and D30. These results suggest an inhibitory role of the inflammatory response for LILRB2 at the first days of CM and a promoting effect 1 month following the disease. The inverse relation between LILRB2 gene expression and IL-8 plasma levels in UM patients also suggests that LILRB2 expression may be involved in the limitation of malaria severity since this chemokine was found to be a risk factor for death during CM (18). It would be thus interesting to test whether inhibition of LILRB2 and maybe LILRB1 and Tim3 could help restore the phagocytosis capacity of monocytes during the first days of CM and limit malaria severity.

Although the strength of our study was the characterization of patients’ monocytes with a 30-day follow-up for CM patients, the current study has some limitations. The main limit is the low number of subjects included in this analysis, even though we made sure that the groups were homogenous in terms of age and sex ratio. In addition, the monocytes studied here come from peripheral blood only, with monocytes present in the brain being difficult to study for obvious ethical reasons. Despite these limitations, our study provides a better understanding of the interplay between monocytes and malaria infection in the pathogenicity of CM.

## NeuroCM group members

Dissou Affolabi (Pediatric Department, Calavi Hospital, Calavi, Benin); Daniel Ajzenberg (UMR EpiMaCT, INSERM, Limoges University, France); Nicolas Argy (UMR261 MERIT, Université Paris Cité, IRD, F-75006 Paris, France); Bibiane Biokou (Medical laboratory, Mother and Child University and Hospital Center (CHU-MEL), Cotonou, Benin); Maroufou Jules Alao (Pediatric Department, Mother and Child University and Hospital Center (CHU-MEL), Cotonou, Benin); Linda Ayedadjou (Pediatric Department, Mother and Child University and Hospital Center (CHU-MEL), Cotonou, Benin); Farid Boumediene (UMR EpiMaCT, INSERM, Limoges University, Limoges, France); Josselin Brisset (Infectious Diseases and Tropical Medicine Department, Limoges University Hospital, Limoges, France); Michel Cot (UMR261 MERIT, Université Paris Cité, IRD, F-75006 Paris, France); Jean-Eudes Degbelo (Medical laboratory, Calavi Hospital, Calavi, Benin); Philippe Deloron UMR261 MERIT, Université Paris Cité, IRD, F-75006 Paris, France); Ida Dossou−Dagba (Pediatric Department, Calavi Hospital, Calavi, Benin); Latifou Dramane (Institut de Recherche Clinique du Benin (IRCB), Calavi, Benin); Emilie Guillochon (UMR261 MERIT, Université Paris Cité, IRD, F-75006 Paris, France); Sayeh Jafari-Guemouri (UMR261 MERIT, Université Paris Cité, IRD, F-75006 Paris, France); Valentin Joste (UMR261 MERIT, Université Paris Cité, IRD, F-75006 Paris, France); Claire Kamaliddin (UMR 261 MERIT, Sorbonne Paris Cité University, IRD, Paris, 75006, France); Elisée Kinkpé (Paediatric Department, Calavi Hospital, Calavi, Benin); Anais Labrunie (UMR EpiMaCT, INSERM, Limoges University, Limoges, France); Yélé Ladipo (Pediatric Department, Mother and Child University and Hospital Center (CHU-MEL), Cotonou, Benin); Thomas Lathiere (Ophtalmology department, Limoges University Hospital, Limoges, France); Achille Massougbodji (Institut de Recherche Clinique du Benin (IRCB), Calavi, Benin); Audrey Mowendabeka (Paediatric Department, Mother and Child Hospital, Limoges, France); Jade Papin (UMR261 MERIT, Université Paris Cité, IRD, F-75006 Paris, France); Bernard Pipy (UMR RESTORE, Toulouse 3 University, INSERM, CNRS, EFS, UPS, 31100 Toulouse, France); Pierre-Marie Preux (UMR EpiMaCT, INSERM, IRD, Limoges University, Limoges, France); Marie Raymondeau (UMR EpiMaCT, INSERM, Limoges University, Limoges, France); Darius Sossou (UMR261 MERIT, Université Paris Cité, IRD, F-75006 Paris, France); Brigitte Techer UMR261 MERIT, Université Paris Cité, IRD, F-75006 Paris, France; Laurence Watier (Center for Research in Epidemiology and Population Health (CESP), INSERM U1018, Paris-Saclay University, Montigny-Le-Bretonneux, France).

## Data availability statement

The original contributions presented in the study are included in the article/**Supplementary Materials**, further inquiries can be directed to the corresponding author/s.

## Ethics statement

The studies involving humans were approved by Comité National d’Ethique pour la Recherche en santé of Benin (n°67/MS/DC/SGM/DRFMT/CNERS/SA; 10/17/2017). The studies were conducted in accordance with the local legislation and institutional requirements. Written informed consent for participation in this study was provided by the participants’ legal guardians/next of kin.

## Author contributions

BV: Data curation, Formal Analysis, Investigation, Writing – original draft, Methodology. JR: Data curation, Investigation, Methodology, Writing – review & editing. SD: Methodology, Writing – review & editing. GB: Methodology, Writing – review & editing, Data curation, Investigation. AY: Methodology, Writing – review & editing, Supervision. SH: Supervision, Writing – review & editing, Data curation, Investigation, Validation. JF: Data curation, Investigation, Supervision, Validation, Writing – review & editing. AA: Data curation, Investigation, Supervision, Validation, Writing – review & editing, Conceptualization, Formal Analysis, Funding acquisition, Writing – original draft.

## Supplementary material

Supplementary Figure 1 Analysis of non-opsonic and opsonic phagocytosis of monocytes from UM and CM children according to CFSE MFI levels. Values were compared by the Mann-Whitney U-test, **** P < 0.0001, *** P < 0.001, ** P < 0.01, * P < 0.05.

Supplementary Figure 2 Plasma cytokine levels in children presenting with uncomplicated malaria (UM) and cerebral malaria (CM) at inclusion, D3 and D30 post-inclusion. Values were compared by the Mann-Whitney U-test, *** P < 0.0005, ** P < 0.005, * P < 0.05.

Supplementary Figure 3 plasma CXCL9 and IL-8 are related to LILRB2 and Tim3 gene expression in UM patients at inclusion and in CM patients at D3 and D30. The Spearman correlation test was used to evaluate the relation between phagocytosis capacity of monocytes and gene expression level in uncomplicated malaria (UM) and CM groups and during follow-up for the CM group (D3, D30). Correlations between LILRB2 gene expression and plasma CXCL9 levels in CM patients at D3 **(A)** and D30 **(B)**. **(C)** Correlations between Tim3 gene expression and plasma CXCL9 levels in CM patients at D30. **(D)** Correlations between LILRB2 gene expression and plasma IL-8 levels in UM patients at inclusion.
