# Supplementary material for: Monocytes, particularly nonclassical ones, lose their opsonic and nonopsonic phagocytosis capacity during pediatric cerebral malaria
Source: Front Immunol. 2024 May 21;15:1358853. doi: 10.3389/fimmu.2024.1358853 (PMC11148436; doi:10.3389/fimmu.2024.1358853)
Supplement: Supplementary file 4 [file Table_1.docx]

**Supplementary Table 1:** Forward and reverse primer sequences used for determination of monocyte transcript levels

| **Gènes** | **Forward sequences (5’->3’)** | **Reverse sequences (5’->3’)** |
| --- | --- | --- |
| **CD64 (FcγRI)** | ATACAGGTGCCAGAGAGGTCTC | CCAGCTTATCCTTCCACGCATG |
| **CD16a (FcγRIIIA)** | GGTGACTTGTCCACTCCAGTGT | ACCATTGAGGCTCCAGGAACAC |
| **CR1** | TAGGTGTCAGCCTGGCTTTGTC | GACATCTGGAGGTGGCTGACAT |
| **CR3** | GGAACGCCATTGTCTGCTTTCG | ATGCTGAGGTCATCCTGGCAGA |
| **CD163** | CCAGAAGGAACTTGTAGCCACAG | CAGGCACCAAGCGTTTTGAGCT |
| **CD36** | CAGGTCAACCTATTGGTCAAGCC | GCCTTCTCATCACCAATGGTCC |
| **CD206** | AGCCAACACCAGCTCCTCAAGA | CAAAACGCTCGCGCATTGTCCA |
| **TLR2** | CTTCACTCAGGAGCAGCAAGCA | ACACCAGTGCTGTCCTGTGACA |
| **LILRB1** | CTCCCTATGAGTGGTCTCTACC | CTGTTGTAGCCAGCATCAGAGC |
| **LILRB2** | GTGTGGTCTTCACCCAGTGATC | AGCCGACATCAGAGACACACTG |
| **Tim3** | GACTCTAGCAGACAGTGGGATC | GGTGGTAAGCATCCTTGGAAAGG |
| **Beta-actin** | GTGGCCGAGGACTTTGATT | ATGCTATCACCTCCCCTGTG |

Primers sequences were designed on Origene website and ordered from IDT, France.
